# Fração de Ejeção do Ventrículo Esquerdo Aumentada, Diminuída ou Estável ao Longo do Tempo em uma Série de 626 Pacientes com Insuficiência Cardíaca que Receberam Tratamento Médico

**DOI:** 10.36660/abc.20200250

**Published:** 2021-07-21

**Authors:** Meng-Meng Han, Wen-Shu Zhao, Xiao-Rong Xu, Xin Wang, Kui-Bao Li, Cai-Jing Dang, Juan Zhang, Jia-Mei Liu, Mu-Lei Chen, Xin-Chun Yang, Lin Xu, Hua Wang

**Affiliations:** 1 Beijing Longfu Hospital Intensive Care Unit Beijing China Intensive Care Unit, Beijing Longfu Hospital, Beijing - China; 2 Capital Medical University Beijing Chaoyang Hospital Heart Center and Beijing Key Laboratory of Hypertension Research Beijing China Heart Center and Beijing Key Laboratory of Hypertension Research, Beijing Chaoyang Hospital, Capital Medical University, Beijing - China

**Keywords:** Insuficiência Cardíaca/mortalidade, Volume Sistólico, Disfunção Ventricular Esquerda, Prognóstico, Uso de Medicamentos

## Abstract

**Fundamento:**

A fração de ejeção (FE) tem sido utilizada em análises fenotípicas e na tomada de decisões sobre o tratamento de insuficiência cardíaca (IC). Assim, a FE tornou-se parte fundamental da prática clínica diária.

**Objetivo:**

Este estudo tem como objetivo investigar características, preditores e desfechos associados a alterações da FE em pacientes com diferentes tipos de IC grave.

**Métodos:**

Foram incluídos neste estudo 626 pacientes com IC grave e classe III–IV da New York Heart Association (NYHA). Os pacientes foram classificados em três grupos de acordo com as alterações da FE, ou seja, FE aumentada (FE-A), definida como aumento da FE ≥10%, FE diminuída (FE-D), definida como diminuição da FE ≥10%, e FE estável (FE-E), definida como alteração da FE <10%. Valores p inferiores a 0,05 foram considerados significativos.

**Resultados:**

Dos 377 pacientes com IC grave, 23,3% apresentaram FE-A, 59,5% apresentaram FE-E e 17,2% apresentaram FE-D. Os resultados mostraram ainda 68,2% de insuficiência cardíaca com fração de ejeção reduzida (ICFEr) no grupo FE-A e 64,6% de insuficiência cardíaca com fração de ejeção preservada (ICFEp) no grupo FE-D. Os preditores de FE-A identificados foram faixa etária mais jovem, ausência de diabetes e fração de ejeção do ventrículo esquerdo (FEVE) menor. Já os preditores de FE-D encontrados foram ausência de fibrilação atrial, baixos níveis de ácido úrico e maior FEVE. Em um seguimento mediano de 40 meses, 44,8% dos pacientes foram vítimas de morte por todas as causas.

**Conclusão:**

Na IC grave, a ICFEr apresentou maior percentual no grupo FE-A e a ICFEp foi mais comum no grupo FE-D.

## Introdução

A fração de ejeção (FE) tem sido utilizada em análises fenotípicas e na tomada de decisões sobre o tratamento de insuficiência cardíaca (IC).^1^ Assim, a FE tornou-se parte fundamental da prática clínica diária. A IC é atualmente classificada de acordo com a FE — insuficiência cardíaca com fração de ejeção reduzida (ICFEr; FE<40%), insuficiência cardíaca com fração de ejeção intermediária (ICFEi; FE 40–49%) ou insuficiência cardíaca com fração de ejeção preservada (ICFEp; FE≥50%).^2^ A avaliação da FE de referência em todos os pacientes com IC é essencial para o diagnóstico, tratamento e prognóstico. O grau de ativação neuro-humoral e a resposta ao tratamento médico diferem entre os tipos de IC.^3^^–^^5^ Indicações para o tratamento de IC podem surgir com a deterioração da FE.^6^^,^^7^ Além disso, a FE não é uma medida estática e alterações ao longo do tempo são comuns em todos os grupos de IC.^8^^–^^10^

A maioria dos estudos recentes sobre IC incluiu pacientes com classificação funcional II–IV da New York Heart Association (classe NYHA). Entretanto, algumas pesquisas têm se concentrado em pacientes críticos com classe III–IV da NYHA. Adicionalmente, essas pesquisas avaliaram o espectro completo de alterações da FE para todos os grupos de IC, mas não detalharam os determinantes da mudança e o prognóstico associado em pacientes com IC grave. Assim, este estudo examinou os padrões de alteração longitudinal da FE em uma coorte de pacientes com IC grave e analisou se as alterações da FE tiveram implicações prognósticas na ICFEr, ICFEi e ICFEp.

## Métodos

### População do estudo

Pacientes acometidos de IC grave foram incluídos neste estudo entre janeiro de 2011 e dezembro de 2016. Os cardiologistas responsáveis chegaram ao diagnóstico de IC com base nos critérios do estudo de Framingham.^11^ A gravidade da IC foi definida como classe III–IV da NYHA, N-terminal do pró-peptídeo natriurético cerebral (NT-proBNP) >1000 pg/mL e teste de caminhada de 6 minutos <150 m. Pacientes com tipos diferentes de IC foram classificados de acordo com as novas Diretrizes da Sociedade Europeia de Cardiologia (*European Society of Cardiology* — ESC) para o diagnóstico e tratamento da IC crônica e aguda: ICFEr, ICFEi e ICFEp. Esta pesquisa foi aprovada pelo Comitê de Ética do Beijing Chaoyang Hospital, Capital Medical University, e foi conduzida em conformidade com a declaração de Helsinki. O termo de consentimento livre e esclarecido foi obtido de todos os participantes.

### Critérios de inclusão e exclusão

**Figura 1:**
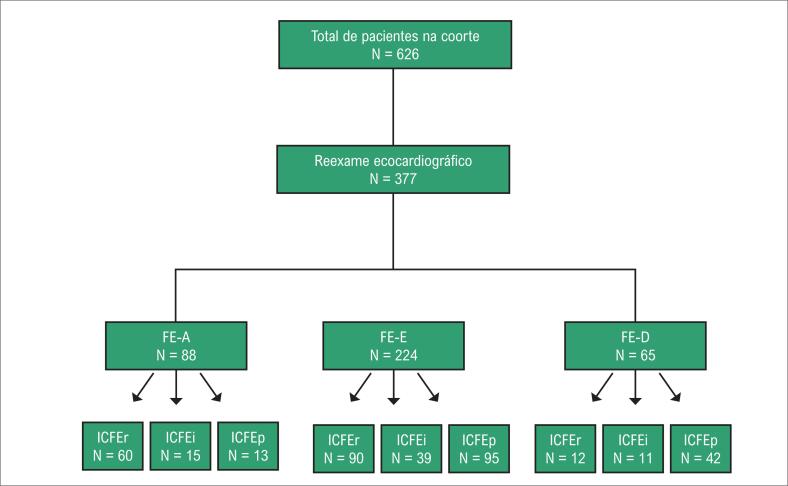
Fluxograma de pacientes incluídos no estudo. FE-A: fração de ejeção aumentada; FE-E: fração de ejeção estável; FE-D: fração de ejeção diminuída; ICFEr: insuficiência cardíaca com fração de ejeção reduzida; ICFEi: insuficiência cardíaca com fração de ejeção intermediária; ICFEp: insuficiência cardíaca com fração de ejeção preservada.

Critérios de inclusão: (1) pacientes com IC grave e classe III–IV da NYHA; (2) pacientes com dados completos de histórico clínico e anamnese; (3) pacientes com 18 anos ou mais. Critérios de exclusão: (1) pacientes com dispneia não cardíaca; (2) pacientes com choque cardiogênico; (3) pacientes com infarto agudo do miocárdio; (4) pacientes com doenças terminais e tempo de sobrevida previsto <1 ano (por exemplo, câncer terminal); (5) pacientes grávidas ou lactantes (Figura 1).

### Coleta de dados

Todas as informações dos pacientes, incluindo características demográficas, anamnese, exames laboratoriais, resultados ecocardiográficos e uso de medicação, foram coletadas a partir dos prontuários eletrônicos por um único pesquisador como dados de referência.

**Figura 2:**
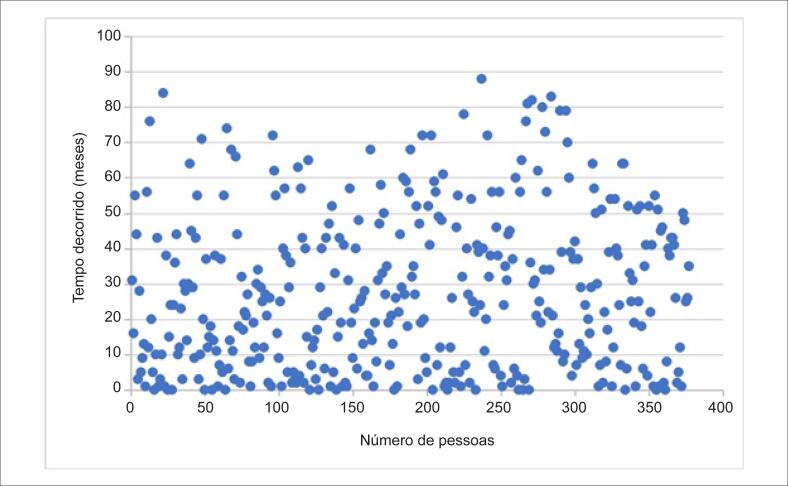
Gráfico de dispersão da distribuição do tempo decorrido entre os exames de ultrassom.

Pacientes com pelo menos duas avaliações de FE foram incluídos neste estudo. Para pacientes com mais de duas avaliações de FE, foram considerados o primeiro e o último resultado para o cálculo de alteração da FE. O tempo decorrido entre os dois exames foi demonstrado por meio de um gráfico de dispersão (Figura 2). Um ultrassonografista cardíaco realizou todos os estudos ecocardiográficos utilizando um aparelho de ultrassom VV5. Técnicas padrão foram adotadas para obtenção de medidas de modo M, bidimensionais e de Doppler conforme as diretrizes da Sociedade Americana de Ecocardiografia.^12^ Os pacientes foram divididos em três grupos com base nas alterações da FE: FE aumentada (FE-A), definida como aumento da FE ≥10%, FE diminuída (FE-D), definida como diminuição da FE ≥10% e FE estável (FE-E), definida como alteração da FE <10%. O método utilizado para calcular a taxa de filtração glomerular estimada (TFGe) foi a modificação da dieta na doença renal (*Modification of Diet in Renal Disease* — MDRD) e para medir o NT-proBNP foi a eletroluminescência.

Todos os pacientes foram acompanhados por telefone ou em ambulatórios e os desfechos primários foram registrados. Os desfechos primários incluíram morte por todas as causas.

### Análise estatística

O programa SPSS 22.0 foi utilizado para a realização da análise estatística. A distribuição normal foi testada nas variáveis contínuas pelo método de Kolmogorov-Smirnov. As variáveis contínuas foram expressas como média±desvio padrão (DP) ou mediana com intervalo interquartil (IIQ) segundo o teste de normalidade. As variáveis categóricas foram expressas como porcentagem (%). Para comparações múltiplas, cada valor foi comparado por análise de variância (ANOVA) de uma via, seguindo o teste de Dunnett, quando os dados apresentavam distribuição normal, enquanto os dados contínuos com distribuição não normal foram comparados por testes não paramétricos (teste H de Kruskal-Wallis). Variáveis categóricas foram testadas pelo teste qui-quadrado. Modelos de regressão logística multivariada foram aplicados para avaliar preditores independentes de FE aumentada ou diminuída. Como os preditores de alteração da FE eram desconhecidos na maioria dos casos, os modelos multivariados incluíram todas as variáveis clinicamente relevantes que poderiam potencialmente afetar a FE ao longo do tempo. Curvas de Kaplan-Meier foram utilizadas para avaliar a associação entre as alterações da FE e a morte por todas as causas e comparadas pelo teste de Mantel-Cox. Valores p<0,05 foram considerados estatisticamente significativos.

## Resultados

### Dados gerais

**Figura 3:**
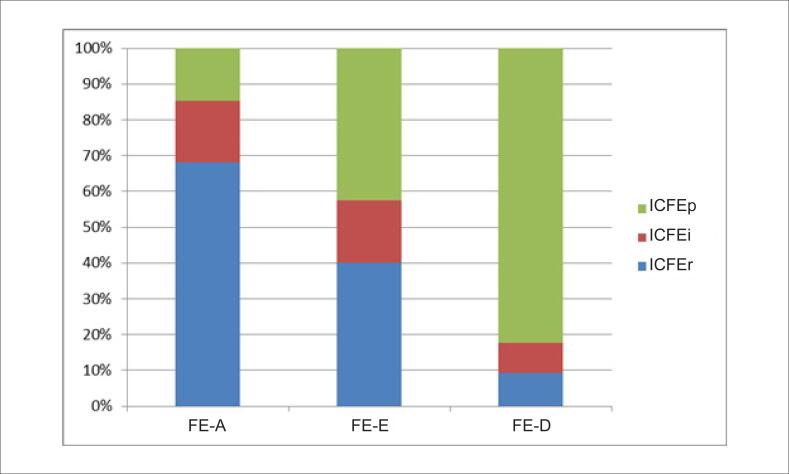
Tipos diferentes de insuficiência cardíaca de acordo com mudanças na fração de ejeção. FE-A: fração de ejeção aumentada; FE-E: fração de ejeção estável; FE-D: fração de ejeção diminuída; ICFEr: insuficiência cardíaca com fração de ejeção reduzida; ICFEi: insuficiência cardíaca com fração de ejeção intermediária; ICFEp: insuficiência cardíaca com fração de ejeção preservada.

Foram incluídos neste estudo 626 pacientes com IC grave e classe III–IV da NYHA entre janeiro de 2011 e dezembro de 2016. Após uma mediana de 27 meses, 377 pacientes com pelo menos 2 exames ecocardiográficos foram inseridos nesta análise. As informações dos pacientes são descritas na Figura 1. O perfil geral da população foi: média de idade de 67±13 anos, 60,2% do sexo masculino, 43,0% com ICFEr, 17,2% com ICFEi e 39,8% com ICFEp. De acordo com a primeira medida do Doppler, 88 pacientes (23,3%) apresentavam FE-A — 68,2% com ICFEr, 17,0% com ICFEi e 14,8% com ICFEp; 224 pacientes (59,5%) apresentavam FE-E — 40,2% com ICFEr, 17,4% com ICFEi e 42,4% com ICFEp; e 65 pacientes (17,2%) apresentavam FE-D — 18,5% com ICFEr, 16,9% com ICFEi e 64,6% com ICFEp (Figura 3).

### Características de referência dos pacientes

Os pacientes com FE-A eram mais jovens e predominantemente do sexo masculino, enquanto os pacientes com FE-D apresentaram maior percentual de cardiopatia isquêmica e ICFEp. Além disso, os pacientes com FE-A tinham maior frequência cardíaca e menor pressão arterial sistólica. A média da pressão arterial sistólica foi 129 mmHg, da pressão arterial diastólica foi 80 mmHg e da frequência cardíaca foi 78 bpm entre os pacientes com ICFEr do grupo FE-A. No entanto, pacientes com FE-D demonstraram níveis mais baixos de hemoglobina e ácido úrico. Não houve diferença de NT-proBNP e TFGe entre os três grupos de alteração da FE. Ademais, os pacientes com FE-A apresentaram níveis menores de FEVE e maior diâmetro diastólico final do ventrículo esquerdo (DDFVE) ou diâmetro sistólico final do ventrículo esquerdo (DSFVE). O uso de digoxina e antagonistas da aldosterona (AA) foi maior entre pacientes com FE-A, enquanto o uso de betabloqueadores, inibidores da enzima conversora da angiotensina/bloqueadores do receptor da angiotensina II (IECA/BRA) e diuréticos de alça não diferiram entre os três grupos (Tabela 1). Um total de 106 pacientes com ICFEr faziam uso de betabloqueadores, 107 de IECA/BRA e 133 de AA. Contudo, a dose desses medicamentos foi difícil de ser coletada, já que os pacientes tomavam tipos diferentes de betabloqueadores e IECA/BRA.

**Tabela 1:** Características de referência de acordo com alterações da fração de ejeção

		FE-A	FE-E	FE-D	Valor p
		(n=88)	(n=224)	(n=65)	
Idade, anos		61,2±13,0	68,8±12,8	70,0±11,8	0,001
Masculino, n (%)		65 (73,9%)	129 (57,6%)	33 (50,8%)	0,007
Etiologia, n (%)					0,014
Isquêmica		43 (48,9%)	139 (62,1%)	45 (69,2%)	
Valvar		8 (9,1%)	33 (14,7%)	9 (13,8%)	
Cardiopatia		21 (23,9%)	25 (11,2%)	4 (6,2%)	
Hipertensão		9 (10,2%)	18 (8,0%)	6 (9,2%)	
Outra		7 (8,0%)	9 (4,0%)	1 (1,5%)	
**Histórico clínico, n (%)**					
	Hipertensão	58 (65,9%)	161 (71,9%)	50 (76,9%)	0,318
	Diabetes mellitus	32 (36,4%)	112 (50,0%)	29 (44,6%)	0,092
	Fibrilação atrial	34 (38,6%)	93 (41,5%)	18 (27,7%)	0,131
	Insuficiência cardíaca prévia	20 (22,7%)	80 (35,7%)	13 (20,0%)	0,012
Tipo de IC, n (%)					0,001
	ICFEr	60 (68,2%)	90 (41,2%)	12 (18,5%)	
	ICFEi	15 (17,0%)	39 (17,4%)	11 (16,9%)	
	ICFEp	13 (14,8%)	95 (42,4%)	42 (64,6%)	
IMC, kg/m^2^		25,2±3,9	24,9±4,5	24,1±4,4	0,371
FC, bpm		89,5±23,6	81,4±19,9	80,9±16,2	0,004
PAS, mmHg		133,3±27,6	134,7±24,1	142,4±23,4	0,052
PAD, mmHg		81,5±16,7	77,3±14,7	80,1±13,4	0,060
Hemoglobina, g/L		131,4±23,6	119,8±21,0	118,8±22,1	0,001
Albumina, g/L		33,8±5,4	34,3±4,9	33,1±4,7	0,211
LDL-C, mmol/L		2,2±0,7	2,1±0,8	2,2±0,8	0,568
PCR-as, mg/L		8,1±6,4	6,7±5,0	7,0±5,0	0,149
	cTnI, ng/mL	0,03 (0,00–0,08)	0,03 (0,00–0,08)	0,04 (0,01–0,14)	0,909
CK-MB, ng/mL		0,8 (0,4–1,8)	0,9 (0,4–1,4)	1,0 (0,7–1,7)	0,488
NT-proBNP, pg/mL		3140 (1420–8345)	3071 (1499–6961)	3866 (1541–10163)	0,439
NUS, mmol/L		8,5±5,0	9,2±5,3	8,3±4,6	0,310
TFGe, mL/min/1,73 m^3^		65,9±29,8	57,3±36,6	56,1±33,0	0,111
AU, umol/L		432,4±126,1	426,1±139,7	371,5±119,9	0,008
Sódio, mmol/L		139,2±3,3	143,6±6,6	138,7±3,5	0,695
Potássio, mmol/L		4,0±0,6	4,0±0,6	4,0±0,6	0,722
Glicemia, mmol/L		5,9±2,8	6,2±2,6	6,5±2,8	0,387
HbA1c, % Ecocardiograma		6,6±1,3	6,7±1,3	6,8±1,3	0,749
FEVE, %		36,8±11,6	47,4±15,7	55,3±14,4	0,001
DDFVE, mm		59,6±8,5	56,5±9,1	53,8±9,3	0,001
DSFVE, mm		48,9±9,7	42,8±11,4	38,3±10,9	0,001
**Medicação, n (%)**					
Digoxina		60 (68,2%)	125 (55,8%)	25 (38,5%)	0,001
Betabloqueadores		56 (63,6%)	126 (56,3%)	41 (63,1%)	0,381
IECA/BRA		57 (64,8%)	117 (52,2%)	34 (52,3%)	0,118
Diuréticos de alça		76 (86,4%)	188 (83,9%)	50 (76,9%)	0,279
AA		70 (79,5%)	160 (71,4%)	39 (60,0%)	0,030

*IMC: índice de massa corporal; FC: frequência cardíaca; PAS: pressão arterial sistólica; PAD: pressão arterial diastólica; LDL-C: colesterol da lipoproteína de baixa densidade; PCR-as: proteína C reativa de alta sensibilidade; cTnI: troponina I cardíaca; CK-MB: isoenzima MB da creatina quinase; NT-proBNP: N-terminal do pró-peptídeo natriurético cerebral; NUS: nitrogênio ureico no sangue; TFGe: taxa de filtração glomerular estimada; AU: ácido úrico; HbA1c: hemoglobina glicada; FEVE: fração de ejeção do ventrículo esquerdo; DDFVE: diâmetro diastólico final do ventrículo esquerdo; DSFVE: diâmetro sistólico final do ventrículo esquerdo; IECA: inibidor da enzima conversora da angiotensina; BRA: bloqueador do receptor de angiotensina II; AA: antagonista da aldosterona.*

### Preditores de fração de ejeção aumentada ou diminuída

A Tabela 2 apresenta a razão de chances (*odds ratio* — OR) ajustada para FE-A e FE-D após a análise de regressão logística multivariada. Faixa etária mais jovem, ausência de diabetes e FEVE menor foram associadas com a FE-A. Por sua vez, as variáveis preditoras de FE-D foram ausência de fibrilação atrial, baixos níveis de ácido úrico e maior FEVE. Nenhum medicamento foi associado à alteração da FE após ajuste para fatores multivariados (Tabela 2).

**Tabela 2:** Associação entre histórico clínico e alteração da fração de ejeção

Variáveis	FE-A		FE-D	
	OR (IC95%)	p	OR (IC95%)	p
Idade (por incremento de 10 anos)	0,677 (0,509–0,902)	0,008		
Diabetes	0,509 (0,285–0,909)	0,022		
Fibrilação atrial			0,430 (0,230–0,805)	0,008
AU (por incremento de 100 umol/L)			0,743 (0,575–0,960)	0,023
FEVE	0,947 (0,926–0,968)	0,001	1,048 (1,028–1,067)	0,001

*FE-A: fração de ejeção aumentada; FE-D: fração de ejeção diminuída; FEVE: fração de ejeção do ventrículo esquerdo.*

### Desfechos clínicos

A mortalidade por todas as causas de acordo com as alterações da FE na IC geral, na ICFEr, na ICFEi e na ICFEp foi: em um seguimento mediano de 40 meses, 44,8% de todos os pacientes do estudo foram vítimas de morte por todas as causas. O grupo FE-A apresentou maior sobrevida que os grupos FE-E e FE-D, embora esse aumento não seja estatisticamente significativo (p=0,064) (Tabela 3).

**Tabela 3:** Mortalidade por todas as causas para alterações da fração de ejeção em diferentes tipos de insuficiência cardíaca

	FE-A	FE-E	FE-D	p
IC	31 (35,2%)	104 (46,4%)	34 (52,3%)	0,064
ICFEr	18 (30,0%)	42 (46,7%)	6 (50,0%)	0,048
ICFEi	6 (40,0%)	17 (43,6%)	5 (45,5%)	0,981
ICFEp	7 (53,8%)	45 (47,4%)	23 (54,8%)	0,976

*FE-A: fração de ejeção aumentada; FE-E: fração de ejeção estável; FE-D: fração de ejeção diminuída; ICFEr: insuficiência cardíaca com fração de ejeção reduzida; ICFEi: insuficiência cardíaca com fração de ejeção intermediária; ICFEp: insuficiência cardíaca com fração de ejeção preservada.*

**Figura 4:**
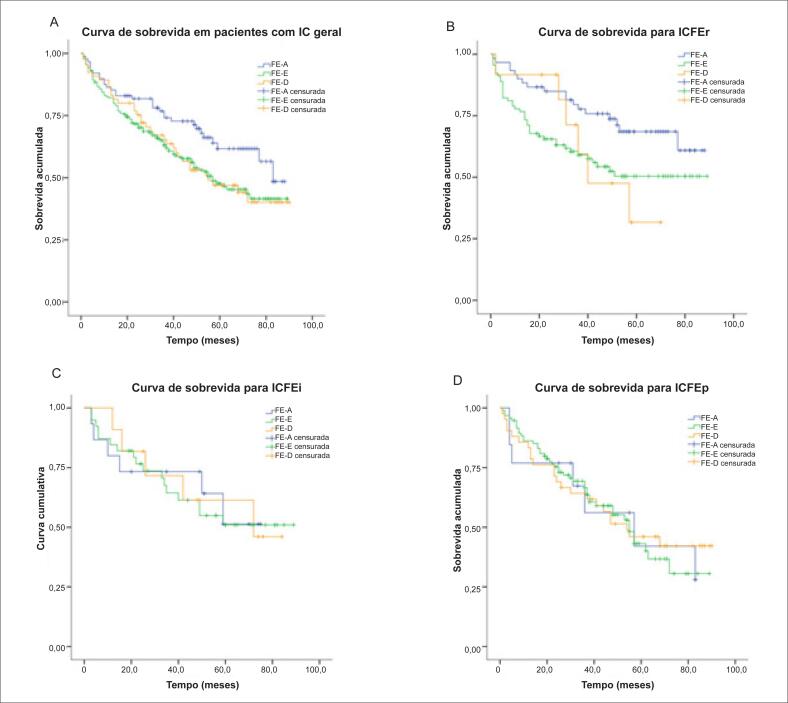
A) Curva de Kaplan-Meier de mortalidade por todas as causas para alteração da fração de ejeção em insuficiência cardíaca geral. B) Curva de Kaplan-Meier de mortalidade por todas as causas para alteração da fração de ejeção em insuficiência cardíaca com fração de ejeção reduzida. C) Curva de Kaplan-Meier de mortalidade por todas as causas para alteração da fração de ejeção em insuficiência cardíaca com fração de ejeção intermediária. D) Curva de Kaplan-Meier de mortalidade por todas as causas para alteração da fração de ejeção em insuficiência cardíaca com fração de ejeção preservada.

Na ICFEr, os pacientes com FE-A apresentaram mortalidade significativamente menor em relação aos com FE-E e FE-D. Ao mesmo tempo, não foram encontradas diferenças na mortalidade por todas as causas relacionadas a alterações da FE na ICFEi e ICFEp. As curvas de Kaplan-Meier estimaram a mortalidade para diferentes tipos de IC, como mostra a Figura 4.

## Discussão

Os resultados deste estudo revelaram que, dos 377 pacientes com IC grave, 23,3% apresentaram FE-A, 59,5% apresentaram FE-E e 17,2% apresentaram FE-D. Adicionalmente, 68,2% dos pacientes com ICFEr estavam no grupo FE-A e 64,6% dos pacientes com ICFEp estavam no grupo FE-D. Os preditores de FE-A identificados foram faixa etária mais jovem, ausência de diabetes e FEVE menor. Já os preditores de FE-D encontrados foram ausência de fibrilação atrial, baixos níveis de ácido úrico e maior FEVE. Em um seguimento mediano de 40 meses, 44,8% dos pacientes foram vítimas de morte por todas as causas. A mortalidade no grupo FE-A foi menor que no grupo FE-D entre os pacientes com ICFEr. Entretanto, não foram encontradas diferenças na mortalidade por todas as causas relacionadas à alteração da FE em pacientes com ICFEi e ICFEp.

### Alteração da fração de ejeção em pacientes com insuficiência cardíaca grave

A IC grave apresenta sintomas e resultados preocupantes quanto à função cardíaca, levando a taxas de mortalidade extremamente altas e prognósticos insatisfatórios. Alguns estudos anteriores investigaram a melhora da FE em pacientes com ICFEr.^6^^,^^7^^,^^13^^–^^15^ Ainda assim, a extensão da alteração da FE em pacientes com IC grave tem sido pouco revisada. Zhang et al.^16^ revelaram que o uso de metoprolol combinado com irbesartana, hidroclorotiazida e ventilação não-invasiva ajudou a recuperar a função cardíaca normal, aumentando o débito cardíaco e normalizando a FE de pacientes com IC grave. Este estudo mostrou que 23,3% dos pacientes com IC grave apresentaram FE-A, 17,2% FE-D e 59,5% FE-E. Dunlay et al.^9^ relataram que uma proporção significativa de pacientes com ICFEp teve redução da FE <50% e uma proporção similar de pacientes com ICFEr teve aumento da FE de ≥50%. A presente pesquisa também identificou que a história natural da IC foi semelhante em pacientes com IC grave.

### Características clínicas e preditores de alteração da fração de ejeção

Entender as características clínicas e os preditores da alteração da FE é essencial, pois eles fornecem informações importantes e podem ser utilizados na estratificação de risco e no direcionamento do tratamento para pacientes com IC grave. Neste estudo, a regressão logística multivariada mostrou que a faixa etária mais jovem, a ausência de diabetes e a FEVE menor estavam associadas à FE-A. Por sua vez, as variáveis preditoras da FE-D foram ausência de fibrilação atrial, baixos níveis de ácido úrico e FEVE mais elevada.

Estudos anteriores relataram que pacientes com melhora da FE eram mais jovens.^9^ No entanto, esta análise identificou a ausência de diabetes como preditora da FE-A, o que pode ser explicado por estudos prévios que detectaram uma associação inversa da diabetes mellitus, da cardiopatia isquêmica e da prescrição de AA na alta hospitalar com a IC e a melhora da FE.^17^^–^^19^

O preditor ausência de fibrilação atrial demonstrou relação com a FE-D, em concordância com um relatório que indicou a fibrilação atrial como um preditor positivo para a melhora da FE. Surpreendentemente, o baixo nível de ácido úrico foi um preditor da FE-D, o que é difícil de explicar.

O presente estudo constatou que a FEVE menor estava associada à FE-A, enquanto a FEVE maior estava associada à FE-D. Dunlay et al.^9^ também identificaram esse resultado em sua análise, já que a FE diminuiu 5,8% em pacientes com ICFEp. Por outro lado, a FE aumentou 6,9% em pacientes com ICFEr ao longo de 5 anos.

### Alteração da fração de ejeção e desfechos nos diferentes tipos de insuficiência cardíaca

Pouco se sabe, a partir de estudos publicados, sobre a implicação prognóstica da alteração da FE em diferentes tipos de IC grave. Este estudo demonstrou que o grupo FE-A teve maior sobrevida que os grupos FE-E e FE-D, embora esse aumento não seja estatisticamente significativo.

No grupo ICFEr, a alteração da FE foi inversamente associada à mortalidade por todas as causas, o que é consistente com achados de estudos anteriores indicando que a recuperação da FEVE reduzida está relacionada a melhores desfechos.^8^^,^^10^ Lupon et al.^6^ observaram que ao utilizar como referência o grupo com recuperação da IC, a razão de risco (*hazard ratio* — HR) da morte cardiovascular ou da hospitalização por IC foi de 2,33 para ICFEp e 1,99 para ICFEr. Essas linhas de evidência sugerem fortemente que a recuperação da ICFEr estava associada a um melhor prognóstico em pacientes com IC. Assim, ressalta-se a importância do controle direcionado a aumentar a FE em pacientes com ICFEr.

A presente pesquisa identificou que as implicações prognósticas da alteração da FE entre os pacientes com ICFEi e ICFEp foram menos evidentes. A falta de associações de risco com a alteração da FE na ICFEi e ICFEp pode resultar da falta de relação linear entre FE e os desfechos da IC quando a FE é mais elevada.

### Destaques deste estudo

Pesquisas anteriores sobre a IC avaliaram pacientes com classe II–IV da NYHA, enquanto este estudo se concentrou em pacientes críticos com classe III–IV da NYHA. Além disso, outras investigações relatam que o prognóstico melhora com o aumento do valor da FE. Adicionalmente, o presente estudo constatou que, em pacientes com IC grave e ICFEr, a FE aumentou após o tratamento, sugerindo assim que o prognóstico pode ser melhorado. Os outros dois tipos de pacientes com IC não foram associados à alteração e ao prognóstico da FE após o tratamento. Os conteúdos mencionados acima foram os destaques deste artigo.

### Limitações

Este estudo apresenta algumas limitações. Primeiro, a amostra, especialmente no grupo FE-D, foi relativamente pequena, o que pode reduzir as diferenças estatisticamente significativas entre esses pacientes. Segundo, este estudo é uma análise retrospectiva de centro único; os achados podem não ser generalizáveis a outras coortes. Terceiro, os pacientes tinham ao menos dois ecocardiogramas para a medida inicial e final da FE, com mediana de 27 meses. No entanto, o ecocardiograma no seguimento de 1 ano não foi registrado, o que poderia ter fornecido novos resultados. Por último, não se chegou a um consenso sobre a definição adequada de alteração da FE. Sabe-se que pequenas melhoras na FEVE, mas ainda qualificadas como FE-A, têm implicações prognósticas prováveis e diferentes do que as maiores. Portanto, para quantificar esse efeito, o presente estudo definiu FE-A como um aumento da FE ≥10%, a FE-D como uma diminuição da FE ≥10% e a FE-E como uma alteração da FE <10%, além da transição para outros fenótipos de IC.

## Conclusões

Na IC grave, a ICFEr apresentou maior percentual no grupo FE-A e a ICFEp foi mais comum no grupo FE-D. A alteração da FE foi associada a uma série de características clínicas, preditores e desfechos, particularmente em pacientes com ICFEr.
