# Supplementary material for: Fração de Ejeção do Ventrículo Esquerdo Aumentada, Diminuída ou Estável ao Longo do Tempo em uma Série de 626 Pacientes com Insuficiência Cardíaca que Receberam Tratamento Médico
Source: Arq Bras Cardiol. 2021 Jul 21;117(4):639–47. [Article in Portuguese] doi: 10.36660/abc.20200250 (PMC8528370; doi:10.36660/abc.20200250)
Supplement: Translation

# Translation: Increased, Decreased, or Stable Left Ventricle Ejection Fraction over Time in a Series of 626 Heart Failure Patients Receiving Medical Treatment

**Authors:** Meng-Meng Han, Wen-Shu Zhao, Xiao-Rong Xu, Xin Wang, Kui-Bao Li, Cai-Jing Dang, Juan Zhang, Jia-Mei Liu, Mu-Lei Chen, Xin-Chun Yang, Lin Xu, Hua Wang

**Affiliations:** 1 Beijing China Intensive Care Unit, Beijing Longfu Hospital, Beijing - China; 2 Beijing China Heart Center and Beijing Key Laboratory of Hypertension Research, Beijing Chaoyang Hospital, Capital Medical University, Beijing - China

**Keywords:** Heart Failure/mortality, Stroke Volume, Ventricular Dysfunction, Left, Prognosis, Drug Utilization

## Abstract

**Background:**

Ejection fraction (EF) has been used in phenotype analyses and to make treatment decisions regarding heart failure (HF). Thus, EF has become a fundamental part of daily clinical practice.

**Objective:**

This study aims to investigate the characteristics, predictors, and outcomes associated with EF changes in patients with different types of severe HF.

**Methods:**

A total of 626 severe HF patients with New York Heart Association (NYHA) class III–IV were enrolled in this study. The patients were classified into three groups according to EF changes, namely, increased EF (EF-I), defined as an EF increase ≥10%, decreased EF (EF-D), defined as an EF decrease ≥10%, and stable EF (EF-S), defined as an EF change <10%. A p-value lower than 0.05 was considered significant.

**Results:**

Out of 377 severe HF patients, 23.3% presented EF-I, 59.5% presented EF-S, and 17.2% presented EF-D. The results further showed 68.2% of heart failure with reduced ejection fraction (HFrEF) in the EF-I group and 64.6% of heart failure with preserved ejection fraction (HFpEF) in the EF-D group. The predictors of EF-I included younger age, absence of diabetes, and lower left ventricular ejection fraction (LVEF). The predictors of EF-D were absence of atrial fibrillation, lower uric acid level, and higher LVEF. Within a median follow-up of 40 months, 44.8% of patients suffered from all-cause death.

**Conclusion:**

In severe HF, HFrEF presented the highest percentage in the EF-I group, and HFpEF was most common in the EF-D group.

## Introduction

Ejection fraction (EF) has been used in phenotype analyses and to make treatment decisions regarding heart failure (HF).^1^ Thus, EF has become a fundamental part of daily clinical practice. HF is currently classified according to EF — heart failure with reduced ejection fraction (HFrEF; EF<40%), heart failure with mid-range ejection fraction (HFmrEF; EF 40–49%), or heart failure with preserved ejection fraction (HFpEF; EF≥50%).^2^ Assessing the baseline EF in all HF patients is essential for diagnosis, treatment, and prognosis. The degree of neurohumoral activation and the response to medical therapy differ among the HF types.^3^^–^^5^ Indications for HF treatment may arise with deteriorating EF.^6^^,^^7^ Moreover, EF is not a static measurement, and changes over time are common in all HF groups.^8^^–^^10^

Most of the recent HF studies have included patients with New York Heart Association functional classification (NYHA class) II–IV. However, a few investigations have focused on critically ill patients with NYHA class III–IV. Furthermore, these studies evaluated the whole spectrum of EF changes for all HF groups but did not detail determinants of change and the associated prognosis in patients with severe HF. Thus, we examined patterns of longitudinal EF change in a cohort of severe HF patients and investigated whether EF changes had prognostic implications in HFrEF, HFmrEF, and HFpEF.

## Methods

### Study population

Patients affected by severe HF were enrolled in this study from January 2011 to December 2016. The attending cardiologists made the diagnosis of HF based on the Framingham study criteria.^11^ HF severity was defined as NYHA class III–IV, N-terminal prohormone of brain natriuretic peptide (NT-proBNP)>1000 pg/mL, and 6-minute walk test<150 m. Patients with different types of HF were classified following the new European Society of Cardiology (ESC) Guidelines for the diagnosis and treatment of chronic and acute HF: HFrEF, HFmrEF, and HFpEF. This study was approved by the Ethics Committee of the Beijing Chaoyang Hospital, Capital Medical University, and complied with the declaration of Helsinki. Written informed consent was obtained from all participants.

### Inclusion and exclusion criteria

Inclusion criteria: (1) patients with severe HF and NYHA class III–IV; (2) patients with complete clinical and medical history data; (3) patients aged 18 years and older. Exclusion criteria: (1) patients with non-cardiac dyspnea; (2) patients with cardiogenic shock; (3) patients with acute myocardial infarction; (4) patients with terminal diseases and predicted survival time<1 year (e.g., terminal cancer); (5) pregnant or lactating patients (Figure 1).

**Figure 1:** Flowchart of patients included in the study. EF-I: increased ejection fraction; EF-S: stable ejection fraction; EF-D: decreased ejection fraction; HFrEF: heart failure with reduced ejection fraction; HFmrEF: heart failure with mid-range ejection fraction; HFpEF: heart failure with preserved ejection fraction.

### Data collection

All patient information, including demographic characteristics, medical history, laboratory tests, echocardiography results, and medication use, was collected from electronic medical records by a single investigator as baseline data.

Patients with at least two EF assessments were enrolled in this study. When the same patient presented more than two EF assessments, the first and last results were considered to calculate the EF change. The time elapsed between the two examinations was shown through a scatter plot (Figure 2). A cardiac sonographer performed all echocardiographic studies using a VV5 ultrasound machine. Standard techniques were adopted to obtain M-mode, 2-dimensional, and Doppler measurements in accordance with the American Society of Echocardiography guidelines.^12^ Patients were divided into three groups based on EF change: increased EF (EF-I), defined as an EF increase ≥10%, decreased EF (EF-D), defined as an EF decrease ≥10%, and stable EF (EF-S), defined as EF change <10%. The method used to calculate the estimated glomerular filtration rate (eGFR) was the Modification of Diet in Renal Disease (MDRD) and to measure NT-proBNP was electroluminescence.

**Figure 2:** Scatter plot of the distribution of time elapsed between the ultrasound examinations.

All patients were followed up by telephone or in outpatient clinics, and the primary endpoints were recorded. The primary endpoints included all-cause death.

### Statistical analysis

We used the SPSS 22.0 software to conduct the statistical analysis. Continuous variables were tested for normal distribution using the Kolmogorov-Smirnov method. Continuous variables were expressed as mean±standard deviation (SD) or median with interquartile range (IQR) according to the normality test. Categorical variables were expressed as a percentage (%). For multiple comparisons, every value was compared by one-way analysis of variance (ANOVA) following the Dunnett test when each datum conformed to a normal distribution, while non-normally distributed continuous data were compared by non-parametric tests (Kruskal-Wallis H test). Categorical variables were tested by the chi-square test. Multivariate logistic regression models were applied to assess independent predictors of EF increase or decrease. Since the predictors of EF change were mostly unknown, multivariate models included all clinically relevant variables that could potentially affect EF over time. Kaplan-Meier curves were used to evaluate the association between EF changes and all-cause death and were compared by a log-rank test. A p-value<0.05 was considered statistically significant.

## Results

### General data

A total of 626 severe HF patients with NYHA class III–IV were enrolled in this study from January 2011 to December 2016. After a median of 27 months, 377 patients with at least 2 echocardiographic examinations were included in our analysis. Patient information is shown in Figure 1. The overall population profile was: mean age of 67±13 years, 60.2% of males, 43.0% with HFrEF, 17.2% with HFmrEF, and 39.8% with HFpEF. According to the first Doppler measurement, 88 patients (23.3%) presented EF-I — 68.2% HFrEF, 17.0% HFmrEF, and 14.8% HFpEF; 224 patients (59.5%) presented EF-S — 40.2% HFrEF, 17.4% HFmrEF, and 42.4% HFpEF; and 65 patients (17.2%) presented EF-D — 18.5% HFrEF, 16.9% HFmrEF, and 64.6% HFpEF (Figure 3).

**Figure 3:** Different types of heart failure according to changes in ejection fraction. EF-I: increased ejection fraction; EF-S: stable ejection fraction; EF-D: decreased ejection fraction; HFrEF: heart failure with reduced ejection fraction; HFmrEF: heart failure with mid-range ejection fraction; HFpEF: heart failure with preserved ejection fraction.

### Baseline patient characteristics

Patients with EF-I were younger and mostly male, whereas patients with EF-D had a higher percentage of ischemic heart disease and HFpEF. Also, patients with EF-I had a higher heart rate and lower systolic blood pressure. The mean systolic blood pressure was 129 mmHg, diastolic blood pressure was 80 mmHg, and heart rate was 78 bpm among patients with HFrEF within the EF-I group. However, patients with EF-D had lower hemoglobin and uric acid levels. No difference was found in NT-proBNP and eGFR among the three EF change groups. In addition, patients with EF-I showed lower LVEF levels and larger left ventricular end-diastolic diameter (LVEDD) or left ventricular end-systolic diameter (LVESD). The use of digoxin and mineralocorticoid receptor antagonists (MRA) was higher among patients with EF-I, while the use of β-blockers, angiotensin-converting enzyme inhibitor/angiotensin II receptor blocker (ACEI/ARB), and loop diuretics did not differ among the three groups (Table 1). A total of 106 patients with HFrEF were on β-blockers, 107 were on ACEI/ARBs, and 133 were on MRAs. However, the dose of these medications was hard to collect as the patients took different kinds of β-blockers and ACEI/ARBs.

**Table 1:** Baseline characteristics according to ejection fraction changes

		EF-I	EF-S	EF-D	P-value
		(n=88)	(n=224)	(n=65)	
Age, years		61.2±13.0	68.8±12.8	70.0±11.8	0.001
Male, n (%)		65 (73.9%)	129 (57.6%)	33 (50.8%)	0.007
Etiology, n (%)					0.014
Ischemic		43 (48.9%)	139 (62.1%)	45 (69.2%)	
Valvular		8 (9.1%)	33 (14.7%)	9 (13.8%)	
Cardiopathy		21 (23.9%)	25 (11.2%)	4 (6.2%)	
Hypertension		9 (10.2%)	18 (8.0%)	6 (9.2%)	
Other		7 (8.0%)	9 (4.0%)	1 (1.5%)	
**Clinical history, n (%)**					
	Hypertension	58 (65.9%)	161 (71.9%)	50 (76.9%)	0.318
	Diabetes mellitus	32 (36.4%)	112 (50.0%)	29 (44.6%)	0.092
	Atrial fibrillation	34 (38.6%)	93 (41.5%)	18 (27.7%)	0.131
	Previous heart failure	20 (22.7%)	80 (35.7%)	13 (20.0%)	0.012
**Type of HF, n (%)**					0.001
	HFrEF	60 (68.2%)	90 (41.2%)	12 (18.5%)	
	HFmrEF	15 (17.0%)	39 (17.4%)	11 (16.9%)	
	HFpEF	13 (14.8%)	95 (42.4%)	42 (64.6%)	
BMI, kg/m^2^		25.2±3.9	24.9±4.5	24.1±4.4	0.371
HR, bpm		89.5±23.6	81.4±19.9	80.9±16.2	0.004
SBP, mmHg		133.3±27.6	134.7±24.1	142.4±23.4	0.052
DBP, mmHg		81.5±16.7	77.3±14.7	80.1±13.4	0.060
Hemoglobin, g/L		131.4±23.6	119.8±21.0	118.8±22.1	0.001
Albumin, g/L		33.8±5.4	34.3±4.9	33.1±4.7	0.211
LDL-C, mmol/L		2.2±0.7	2.1±0.8	2.2±0.8	0.568
hs-CRP, mg/L		8.1±6.4	6.7±5.0	7.0±5.0	0.149
cTnI, ng/mL		0.03 (0.00–0.08)	0.03 (0.00–0.08)	0.04 (0.01–0.14)	0.909
CK-MB, ng/mL		0.8 (0.4–1.8)	0.9 (0.4–1.4)	1.0 (0.7–1.7)	0.488
NT-proBNP, pg/mL		3140 (1420–8345)	3071 (1499–6961)	3866 (1541–10163)	0.439
BUN, mmol/L		8.5±5.0	9.2±5.3	8.3±4.6	0.310
eGFR, mL/min/1.73m^3^		65.9±29.8	57.3±36.6	56.1±33.0	0.111
URIC, umol/L		432.4±126.1	426.1±139.7	371.5±119.9	0.008
Sodium, mmol/L		139.2±3.3	143.6±6.6	138.7±3.5	0.695
Potassium, mmol/L		4.0±0.6	4.0±0.6	4.0±0.6	0.722
Blood glucose, mmol/L		5.9±2.8	6.2±2.6	6.5±2.8	0.387
HbA1c, % Echocardiography		6.6±1.3	6.7±1.3	6.8±1.3	0.749
LVEF, %		36.8±11.6	47.4±15.7	55.3±14.4	0.001
LVEDD, mm		59.6±8.5	56.5±9.1	53.8±9.3	0.001
LVESD, mm		48.9±9.7	42.8±11.4	38.3±10.9	0.001
**Medication, n (%)**					
Digoxin		60 (68.2%)	125 (55.8%)	25 (38.5%)	0.001
β-blockers		56 (63.6%)	126 (56.3%)	41 (63.1%)	0.381
ACEI/ARB		57 (64.8%)	117 (52.2%)	34 (52.3%)	0.118
Loop diuretics		76 (86.4%)	188 (83.9%)	50 (76.9%)	0.279
MRA		70 (79.5%)	160 (71.4%)	39 (60.0%)	0.030

*BMI: body mass index; HR: heart rate; SBP: systolic blood pressure; DBP: diastolic blood pressure; LDL-C: low-density lipoprotein cholesterol; hs-CRP: high-sensitivity C-reactive protein; cTnI: cardiac troponin I; CK-MB: creatine-kinase MB isoenzyme; NT-proBNP: N-terminal prohormone brain natriuretic peptide; BUN: blood urea nitrogen; eGFR: estimated glomerular filtration rate; URIC: uric acid; HbA1c: glycated hemoglobin; LVEF: left ventricular ejection fraction; LVEDD: left ventricular end-diastolic diameter; LVESD: left ventricular end-systolic diameter; ACEI: angiotensin-converting enzyme inhibitor; ARB: angiotensin II receptor blocker; MRA: mineralocorticoid receptor antagonists.*

### Predictors of ejection fraction increase and decrease

Table 2 presents the adjusted odds ratio (OR) for EF-I and EF-D after the multivariate logistic regression analysis. Younger age, absence of diabetes, and lower LVEF were independently associated with EF-I. On the other hand, variables predicting EF-D included absence of atrial fibrillation, lower uric acid level, and higher LVEF. No medications were associated with EF change after being adjusted for multivariate factors (Table 2).

**Table 2:** Association between clinical backgrounds and ejection fraction changes

Variables	EF-I		EF-D	
	OR (95%CI)	p	OR (95%CI)	p
Age (per 10-year increase)	0.677 (0.509–0.902)	0.008		
Diabetes	0.509 (0.285–0.909)	0.022		
Atrial fibrillation			0.430 (0.230–0.805)	0.008
URIC (per 100 umol/L increase)			0.743 (0.575–0.960)	0.023
LVEF	0.947 (0.926–0.968)	0.001	1.048 (1.028–1.067)	0.001

*EF-I: increased ejection fraction; EF-D: decreased ejection fraction; LVEF: left ventricular ejection fraction.*

### Clinical outcomes

All-cause mortality according to EF changes in overall HF, HFrEF, HFmrEF, and HFpEF was as follows: within a median follow-up of 40 months, 44.8% of all study patients suffered from all-cause death. The EF-I group had a higher survival rate than the EF-S and EF-D groups, although this increase was not statistically significant (p=0.064) (Table 3).

**Table 3:** All-cause mortality for ejection fraction changes in different types of heart failure

	EF-I	EF-S	EF-D	p
HF	31 (35.2%)	104 (46.4%)	34 (52.3%)	0.064
HFrEF	18 (30.0%)	42 (46.7%)	6 (50.0%)	0.048
HFmrEF	6 (40.0%)	17 (43.6%)	5 (45.5%)	0.981
HFpEF	7 (53.8%)	45 (47.4%)	23 (54.8%)	0.976

*EF-I: increased ejection fraction; EF-S: stable ejection fraction; EF-D: decreased ejection fraction; HFrEF: heart failure with reduced ejection fraction; HFmrEF: heart failure with mid-range ejection fraction; HFpEF: heart failure with preserved ejection fraction.*

In HFrEF, patients with EF-I presented significantly lower mortality compared to those with EF-S and EF-D. At the same time, we found no difference in all-cause mortality related to EF changes in HFmrEF and HFpEF. Kaplan-Meier curves estimated mortality for different types of HF, as shown in Figure 4.

**Figure 4:** A) Kaplan-Meier curve of all-cause mortality for ejection fraction change in overall heart failure. B) Kaplan-Meier curve of all-cause mortality for ejection fraction change in heart failure with reduced ejection fraction. C) Kaplan-Meier curve of all-cause mortality for ejection fraction change in heart failure with mid-range ejection fraction. D) Kaplan-Meier curve of all-cause mortality for ejection fraction change in heart failure with preserved ejection fraction.

## Discussion

The outcomes of this study revealed that, among 377 severe HF patients, 23.3% presented EF-I, 59.5% presented EF-S, and 17.2% presented EF-D. Moreover, 68.2% of patients with HFrEF were in the EF-I group, and 64.6% of those with HFpEF were in the EF-D group. Predictors of EF-I included younger age, absence of diabetes, and lower LVEF. The predictors of EF-D were absence of atrial fibrillation, lower uric acid level, and higher LVEF. Within a median follow-up of 40 months, 44.8% of patients suffered from all-cause death. Mortality in the EF-I group was lower than in the EF-D group among HFrEF patients. However, we found no difference in all-cause mortality related to EF change in HFmrEF and HFpEF patients.

### Ejection fraction change in severe heart failure patients

Severe HF has worrying symptoms and results regarding cardiac function, leading to extremely high mortality rates and poor prognoses. Some previous studies have investigated EF improvement in patients with HFrEF.^6^^,^^7^^,^^13^^–^^15^ Still, the extent of the EF change in severe HF patients has been poorly reviewed. Zhang et al.^16^ revealed that metoprolol combined with irbesartan, hydrochlorothiazide, and non-invasive ventilation helped recover normal cardiac function, increasing cardiac output and normalizing the EF of severe HF patients. Our study showed that 23.3% of severe HF patients had EF-I, 17.2% had EF-D, and 59.5% had EF-S. Dunlay et al.^9^ reported that a significant proportion of patients with HFpEF had a decline in EF of <50%, and a similar proportion of patients with HFrEF experienced an increase in EF of ≥50%. The present research also identified that the natural history of HF was similar in patients with severe HF.

### Clinical characteristics and predictors of ejection fraction change

Understanding the clinical characteristics and predictors of EF change is essential, as they provide important information and can be used for risk stratification and for guiding the therapy for patients with severe HF. In the present study, multivariate logistic regression showed that younger age, absence of diabetes, and the lower LVEF were associated with EF-I. In turn, the variables predicting EF-D were the absence of atrial fibrillation, lower uric acid level, and higher LVEF.

Previous studies have reported patients with EF improvement to be younger.^9^ However, our analysis identified the absence of diabetes as a predictor of EF-I, which could be explained by previous studies that detected an inverse association of diabetes mellitus, ischemic heart disease, and MRA prescription at discharge with HF and improved EF.^17^^–^^19^

The predictor absence of atrial fibrillation was related to EF-D, in accordance with a report indicating that atrial fibrillation was a positive predictor for improving EF. Surprisingly, lower uric acid level was a predictor for EF-D, which is difficult to explain.

Our study found that lower LVEF was associated with EF-I, while higher LVEF was associated with EF-D. Dunlay et al.^9^ also identified this result in their analysis, since EF decreased by 5.8% in HFpEF patients. Conversely, EF increased by 6.9% in HFrEF patients over 5 years.

### Ejection fraction change and outcomes in the different types of heart failure

Little is known from published studies about the prognostic implication of EF change in different types of severe HF. This study showed that the EF-I group had a higher survival rate than the EF-S and EF-D groups, although this increase was not statistically significant.

In the HFrEF group, EF change was inversely associated with all-cause mortality, which is consistent with the findings of previous studies indicating that recovery from reduced LVEF is connected with better outcomes.^8^^,^^10^ Lupon et al.^6^ found that by using the HF-recovered group as a reference, the hazard ratio (HR) of cardiovascular death or HF hospitalization was 2.33 for HFpEF and 1.99 for HFrEF. These lines of evidence strongly suggest that recovery from HFrEF was associated with a better prognosis in HF patients. Thus, we underline the importance of management aiming at increasing EF in HFrEF patients.

The present research identified that the prognostic implications of EF change among HFmrEF and HFpEF patients were less evident. The lack of risk associations with EF change in HFmrEF and HFpEF might result from the lack of a linear relationship between EF and HF outcomes when EF is higher.

### Highlights of this study

Previous HF studies assessed patients with NYHA class II–IV, whereas our study focused on critically ill patients with NYHA class III–IV. Furthermore, other investigations report that the prognosis improves with the increase in the EF value. In addition, the present study found that in patients with severe HF and HFrEF, EF increased after treatment, thus suggesting that the prognosis can be improved. The other two types of HF patients were not associated with EF change and prognosis after receiving treatment. The above-mentioned contents were the highlights of this article.

### Limitations

This study has several limitations. First, the sample, especially in the EF-D group, was relatively small, which may reduce the statistically significant differences among these patients. Second, this study is a single-center and retrospective analysis; the findings might not be generalizable to other cohorts. Third, the patients had at least two echocardiograms for the initial and final EF measurement, with a median of 27 months. However, we did not record the echocardiogram at the 1-year follow-up, which could have provided further results. Finally, no consensus has been reached on the appropriate definition of EF change. We recognize that small improvements in LVEF that still qualify as EF-I have probable and different prognostic implications than larger improvements. Therefore, in order to quantify this effect, we defined EF-I as an EF increase ≥10%, EF-D as an EF decrease ≥10%, and EF-S as an EF change <10%, other than the transition to the other HF phenotypes.

## Conclusions

In severe HF, HFrEF presented the highest percentage in the EF-I group, and HFpEF was more common in the EF-D group. EF change was associated with a series of clinical characteristics, predictors, and outcomes, especially in HFrEF patients.
